# Predicting an individual’s functional connectivity from their structural connectome: Evaluation of evidence, recommendations, and future prospects

**DOI:** 10.1162/netn_a_00400

**Published:** 2024-12-10

**Authors:** Andrew Zalesky, Tabinda Sarwar, Ye Tian, Yuanzhe Liu, B. T. Thomas Yeo, Kotagiri Ramamohanarao

**Affiliations:** Systems Lab, Department of Psychiatry, The University of Melbourne, Victoria, Australia; Department of Biomedical Engineering, The University of Melbourne, Victoria, Australia; School of Computing Technologies, RMIT University, Victoria, Australia; Department of Electrical and Computer Engineering, Center for Sleep & Cognition and N.1 Institute for Health, National University of Singapore, Singapore; Retired Professor, The University of Melbourne, Victoria, Australia

**Keywords:** Connectome, Structure-function coupling, Individual differences, Functional connectivity, Prediction model

## Abstract

Several recent studies have optimized deep neural networks to learn high-dimensional relationships linking structural and functional connectivity across the human connectome. However, the extent to which these models recapitulate individual-specific characteristics of resting-state functional brain networks remains unclear. A core concern relates to whether current individual predictions outperform simple benchmarks such as group averages and null conditions. Here, we consider two measures to statistically evaluate whether functional connectivity predictions capture individual effects. We revisit our previously published functional connectivity predictions for 1,000 healthy adults and provide multiple lines of evidence supporting that our predictions successfully capture subtle individual-specific variation in connectivity. While predicted individual effects are statistically significant and outperform several benchmarks, we find that effect sizes are small (i.e., 8%–11% improvement relative to group-average benchmarks). As such, initial expectations about individual prediction performance expressed by us and others may require moderation. We conclude that individual predictions can significantly outperform appropriate benchmark conditions and we provide several recommendations for future studies in this area. Future studies should statistically assess the individual prediction performance of their models using one of the measures and benchmarks provided here.

## Author Summary

Functional and structural brain networks share considerable overlap in network architecture. However, it remains debated whether deep neural networks can be trained to predict an individual's functional brain network from their structural connectome. We demonstrate that individual variability in functional brain connectivity can be successfully predicted from an individual's connectome, although prediction performance is modest when benchmarked against appropriate null models. We provide recommendations for future studies aiming to evaluate such predictions, specifically considering the impact of Riemann geometry, adjustments for cross-validation induced dependence and standardization. Accurate prediction models will enable extrapolation of functional networks for individuals without empirically acquired functional MRI data or noisy data. They may also facilitate digital simulations of the potential functional consequences arising from pathological changes in the connectome.

## INTRODUCTION

Understanding the complex relationship between structural and functional brain networks is a major goal in neuroscience (Fornito, Zalesky, & Bullmore, 2016; Griffa et al., 2022; Honey et al., 2009; Mišić et al., 2016; Suárez et al., 2020). We developed one of the first deep learning frameworks to predict an individual’s functional connectivity (FC) network from their structural connectome (SC) data (Sarwar et al., 2021). Our individual predictions significantly correlated with empirically measured FC (*r* = 0.5 ± 0.1), and we demonstrated that interindividual variation in our predictions was well matched to empirically measured variation. Our work builds upon seminal work by Li and colleagues presented at a signal and information processing conference (Li et al., 2019).

The ability to accurately predict an individual’s FC from their SC is an important advance. It may enable in silico testing of functional changes resulting from simulated connectome perturbations and pathology. It may also enable extrapolation of FC networks for individuals without empirically acquired functional MRI data or noisy data. However, these tasks cannot be achieved without accurate predictions that capture personalized connectivity effects.

Since the publication of our work, several groups have trained more sophisticated deep neural networks and demonstrated improved prediction accuracy in larger cohorts and across diverse age ranges (see Table 1). For example, using the Graph Nets architecture, Neurdorf and colleagues were the first to surpass our prediction accuracy for both group-averaged and individual FC. They also showed that high centrality regions (i.e., regions with a larger than average number of connections) are particularly important to model performance (Neudorf, Kress, & Borowsky, 2022). Soon after, Yang and colleagues developed a graph autoencoder to learn the joint distribution of SC and FC (Yang et al., 2023). The autoencoder can better preserve the topological structure of predicted networks compared to our model. Using the same connectomes that we mapped for our study, Yang and colleagues reported a considerable improvement in group-average prediction accuracy but noted the difficulty in predicting individual FC. In more recent work, Hong and colleagues trained a graph convolutional network to predict a child’s FC across four age bands (Hong et al., 2023). Remarkably, their model accurately predicted a 6-year-old’s FC based on SC measured at 1 year of age. Other groups have considered the inverse problem of predicting SC from FC (Zhang, Wang, & Zhu, 2022). Li and colleagues trained a graph encoder-decoder to predict FC from SC and demonstrated that their predicted FC matrices preserved differences between different behavioural phenotypes (Li, Mateos, & Zhang, 2021).

**Table 1.** Studies using AI to predict an individual’s brain functional connectivity network from their structural connectome

	*N*	Nodes	Approach	eFC-pFC (Pearson’s *r*)	
				Group	Individual
Li et al. (2019)	1,058	68	Graph convolutional net	≈0.75†	
Sarwar et al. (2021)	10^3^	68	Multilayer perceptron	0.9 ± 0.1	0.5 ± 0.1
Neudorf et al. (2022)	998	66	Graph Nets	0.94	0.69
Benkarim et al. (2022)	326	100	Riemannian optimization*	−	0.804 ± 0.060
Yang et al. (2023)	10^3^	68	Graph autoencoder	0.96	0.572
Hong et al. (2023)	360	90	Graph convolutional net	−	0.716–0.724
Chen et al. (2023)	404	400	Graph convolutional net	0.953	0.715 ± 0.052

*Note*. Prediction accuracy evaluated using Pearson’s correlation coefficient. *N*: sample size; Nodes: number of connectome nodes.

* Not an AI approach.

† Value inferred from plot; exact value not reported. Unclear whether correlation is for group or individual.

Complementing these artificial intelligence (AI) approaches, related work has successfully predicted FC by fitting an individual’s FC matrix, or its eigenvectors, to the eigenvectors of the structural connectome’s Laplacian matrix (Abdelnour et al., 2018; Becker et al., 2018; Benkarim et al., 2022; Cummings et al., 2022; Deslauriers-Gauthier et al., 2020). The Laplacian captures information flow across the connectome resulting from a diffusion process. These non-AI approaches based on spectral graph theory have yielded some of the most accurate individual predictions reported to date.

Despite the burgeoning of studies in this area, the extent to which FC predictions truly capture individual effects has been called to question. Smolders and colleagues recently suggested that several published FC predictions do not exceed chance expectations and/or trivial benchmarks, such as group-average connectivity (Smolders et al., 2023). Many studies, including our own work, demonstrate that interindividual variation in predicted FC is matched in distribution to variation in empirical FC. However, Smolders and colleagues reasonably contend that this match in distribution does not necessarily establish that FC predictions recapitulate individual-specific connectivity effects. They show that adding an adequate amount of noise to the group-average FC matrix yields comparable interindividual variation. Furthermore, they report a negligible association between interindividual variation in predicted FC and interindividual variation in empirical FC, potentially suggesting that current predictions do not capture individual effects. Based on these two findings, they question whether our (Sarwar et al., 2021) and several other prediction models (Benkarim et al., 2022; Neudorf, Kress, & Borowsky, 2022) have learnt a meaningful individual SC-to-FC mapping.

In contrast to this work, Chen and colleagues recently provided new evidence showing that FC predictions can potentially capture individual effects. Correlations between an individual’s predicted FC and (i) that individual’s SC (intraindividual coupling), and (ii) the SC of all other individuals (interindividual coupling) were computed. The authors found that intraindividual coupling was significantly greater than interindividual coupling, indicating that the SC-to-FC mapping learnt by the convolutional network captured individual effects (Chen et al., 2023). However, individual differences in structure-function coupling accounted for a relatively small proportion of the overall variance (i.e., < 5%).

Considering the above contentions in the recent literature (Chen et al., 2023; Deslauriers-Gauthier et al., 2020; Smolders et al., 2023), there is a need to scrutinize previously published FC predictions, including our own work, to determine whether they outperform appropriate benchmark and null conditions. Recommendations and standardized tests also need to be established to simplify the assessment, comparison, and evaluation of individual prediction accuracy results in future studies.

To this end, we revisited our previous FC predictions (Sarwar et al., 2021), aiming to rigorously assess the extent to which they significantly and meaningfully outperform several new and established benchmarks. Here, we provide multiple lines of evidence showing that our mapping from SC to FC captures individual effects and it significantly outperforms the group-average FC benchmark and other null models. However, consistent with recent work (Chen et al., 2023), we find that individual connectivity characteristics captured by current predictive models are small and potentially overshadowed by group effects common to all individuals. We conclude that an individual’s FC *can* be predicted from their SC using deep learning approaches. Although distinguishing individuals from group effects is challenging, recent advances have furnished impressive predictions (Jamison et al., 2024). We provide recommendations for future studies in this area and discuss opportunities to improve prediction accuracy. Our work establishes benchmarks and measures for future studies to evaluate the performance of connectome-based predictions of an individual’s FC.

## RESULTS

We use empirical functional connectivity (eFC) to denote functional connectivity mapped from empirically acquired resting-state functional MRI data. Predicted functional connectivity (pFC) denotes functional connectivity predicted from an individual’s structural connectome (SC). Unless otherwise indicated, pFC, eFC, and SC refer to an individual’s connectivity matrix.

**Figure 1.**
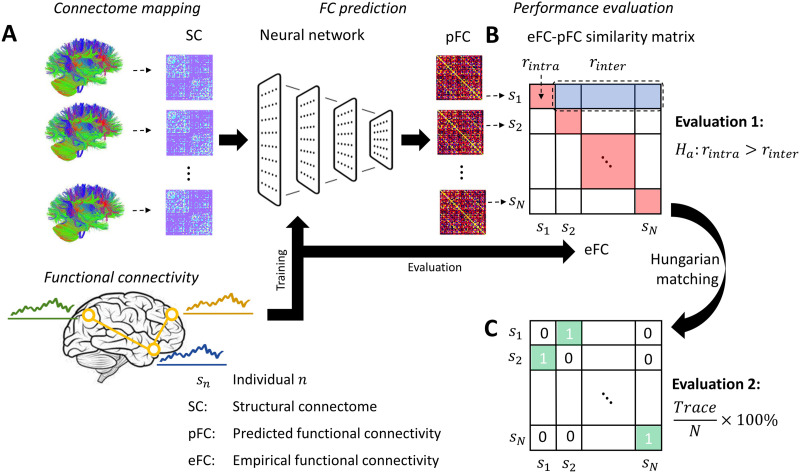
Overview of methodology and performance evaluation. (A) Schematic shows training of a neural network to predict an individual’s functional connectivity matrix from tractography-derived structural connectomes. (B) Diagonal matrix elements quantify intraindividual similarity between predicted (pFC) and empirical (eFC) functional connectivity, denoted *r*_*intra*_ (red cells). Off-diagonal elements quantify interindividual similarity, denoted *r*_*inter*_ (blue cells). Prediction accuracy exceeding chance expectations requires *r*_*intra*_ to significantly exceed the mean of *r*_*inter*_. (C) Matrix shows a one-to-one pFC-to-eFC matching across individuals. The matching is optimized to maximize the sum of similarity values across all nonzero elements of the matrix (green cells). An individual’s pFC would ideally be matched to their eFC, indicated by a 1 on the diagonal. The trace of the binary assignment matrix gives the number of correct matchings.

In this work, we reused our previously established eFC, pFC, and SC matrices for 1,000 healthy young adults participating in the Human Connectome Project (Sarwar et al., 2021). Connectivity matrices encompassed 68 cortical regions comprising the Desikan-Killiany parcellation atlas (Desikan et al., 2006). Constrained spherical deconvolution and whole-brain deterministic tractography was used to map structural connectomes. A multilayer perceptron network was trained to learn the mapping from SC to eFC using 10-fold cross-validation across the 1,000 individuals (see Methods). For each fold, a pFC matrix was predicted for individuals comprising the test fold. A schematic of the workflow is shown in Figure 1A. Further details about connectome mapping and the neural network architecture are available in Sarwar et al. (2021).

Crucially, our customized objective function for neural network training included a regularization function to ensure that interindividual similarity in pFC was matched in distribution to interindividual similarity in eFC (Sarwar et al., 2021). Note that the similarity between two FC matrices was quantified with the elementwise correlation between upper (or lower) triangular elements (see Methods). However, as pointed out by Smolders and colleagues, matching the distributions of interindividual similarity between pFC and eFC does not necessarily imply that predictions capture individual-specific connectivity effects (Smolders et al., 2023). In the present work, we thus considered two new measures to statistically evaluate the extent to which our individual FC predictions (i.e., pFC) capture individual characteristics, as introduced below.

First, we statistically tested whether an individual’s pFC was more similar to their eFC than to the eFC of the remaining *N* − 1 individuals. If true, this would suggest that our predictions capture individual effects that distinguish individuals from others. To test this hypothesis, a similarity matrix, denoted *r*, was computed, where *r*_*ij*_ quantified the elementwise correlation between the pFC matrix for individual *i* and the eFC matrix belonging to individual *j* (Figure 1B). Mean interindividual similarity for individual *i* was given by *r*_*inter*_(*i*) = ∑_*j*≠*i*_ *r*_*ij*_/(*N* − 1), whereas intraindividual similarity was defined as *r*_*intra*_(*i*) = *r*_*ii*_. A one-sample *t* test was used to test the null hypothesis,

$$H_{\text{null}}:\sum_{i}\left(r_{\text{intra}}\left(i\right)-r_{\text{inter}}\left(i\right)\right)/N=0.$$ (1)

Consistent with recent work (Chen et al., 2023), we found that the null hypothesis could be rejected (intra = 0.547, inter = 0.541, *p* = 0.03, *df* = 999), suggesting that our predictions recapitulate individual-specific characteristics of functional connectivity, although the effect size is very small (*d* = 0.06) and corresponds to a 9.1% improvement in *r*_*intra*_ relative to chance levels, calculated as (*r*_*pFC*_ − *r*_*meanFC*_)/*r*_*pFC*_ × 100%, where *r*_*meanFC*_ denotes *r*_*intra*_ in the mean eFC matrix with independent noise (defined below).

The performance of pFC with respect to the above null hypothesis was benchmarked against two distinct control conditions: (i) prediction of the mean eFC matrix (group average) for all individuals, and (ii) prediction of the mean eFC matrix with independent Gaussian noise added to each matrix element (Smolders et al., 2023). Noise standard deviations were matched to deviations observed in eFC. The computation of means and standard deviations for eFC matrix elements respected the cross-validation folds used for neural network training; namely, separate means and standard deviations were computed across individuals comprising each training set. We found that the null hypothesis could not be rejected when substituting an individual’s pFC with either the mean eFC matrix (intra = 0.739, inter = 0.740, *p* = 1.000) or the Gaussian noise null condition (intra = 0.477, inter = 0.477, *p* = 0.706). This suggests that our SC-to-FC mapping can capture individual-specific effects beyond group-average eFC as well as the null condition.

**Figure 2.**
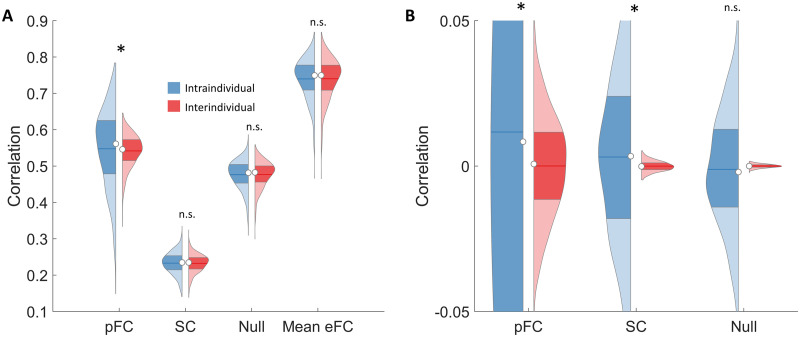
Evaluation of individual prediction accuracy. Violin plots show distributions of intraindividual (blue) and interindividual (red) correlation coefficients (*N* = 1,000). Empirical functional connectivity (eFC) is correlated with predicted functional connectivity (pFC), structural connectome (SC), null connectivity matrices (Null) and the mean eFC matrix computed in the training sample (Mean). (A) Unstandardized pFC and eFC. (B) Z-scored pFC and eFC. *p < 0.05.

Using the same null hypothesis test, we also tested whether an individual’s SC connectome was more similar to their eFC than to the eFC of the remaining individuals. Surprisingly, we found that the null hypothesis could not be rejected when substituting an individual’s pFC with their SC matrix (intra = 0.233, inter = 0.232, *p* = 0.089). Constraining the computation of SC-to-eFC correlations to the set of nonzero SC values for each individual did not alter this result (intra = 0.260, inter = 0.259, *p* = 0.16). This suggests that our SC-to-FC mapping may have unravelled complex individual effects in SC that are not detectable in the SC space with a linear measure such as the correlation coefficient. Using alternative parcellation atlases or improved connectome mapping pipelines may improve individual SC-to-FC coupling, particularly given that a trend toward significance is evident under the current pipeline. The distributions of *r*_*intra*_(*i*) and *r*_*inter*_(*i*), *i* = 1, …, *N* are shown for pFC, SC, mean eFC, and the Gaussian noise null condition in Figure 2A.

### Adjusting for Cross-Validation Induced Dependence

The cross-validation process used to train the neural network can induce dependencies between individual pFC matrices among cross-validation folds. These between-fold dependencies can in turn induce dependencies in the similarity matrix. The independence assumption required of many statistical tests may thus be violated when testing hypotheses involving the similarity matrix. Violation of independence can yield optimistic *p* values (Bouckaert & Frank, 2004), although our use of a paired *t* test may have minimized this effect.

We considered two methods to test our hypothesis while controlling for the dependence induced by cross-validation. First, rather than estimating *p* values parametrically using the Student’s *t* distribution, we used permutation to generate an empirical null distribution for *H*_*null*_. For each permutation, columns of the similarity matrix were permuted. Crucially, permutations were constrained to occur between columns corresponding to individuals assigned to the same cross-validation fold. Permutations were forbidden to occur across different folds, thus ensuring that the null distribution preserved any between-fold dependencies (see Methods and Supporting Information Figure S1A). The above null hypothesis was once again rejected when *p* values were estimated with permutation testing (*p* = 0.0148 ± 0.0034 [95% confidence interval], permutations = 10^4^; Supporting Information Figure S1B), suggesting that dependence minimally affected *p* value estimation.

A corrected resampled *t*-test statistic has been proposed to assess whether the mean difference in accuracy between two prediction models is statistically significant, while controlling for the dependence induced by cross-validation (Bouckaert & Frank, 2004; Nadeau & Bengio, 2003). The mean difference is computed independently for each cross-validation fold. The variance of the mean difference across the *K* folds is then used to define an adjusted standard error for the *t* test (see Methods). Using this approach, we compared the performance of pFC to both benchmark predictions: (i) mean eFC, and, (ii) mean eFC with noise. Specifically, the corrected *t* test was used to test the null hypothesis,

$$H_{\text{null}}:\frac{1}{K}\sum_{k=1,\ldots ,K}f\left(\delta_{pFC}\left(k\right)-\delta_{\text{meanFC}}\left(k\right)\right)=0.$$ (2)

Under this formulation, ***δ***(*k*) = ***r***_*intra*_(*k*) − ***r***_*inter*_(*k*) is an *n* × 1 vector of differences in prediction performance for the *n* individuals in fold *k* = 1, …, *K*, *f*(·) denotes a central tendency measure such as the median or mean. We used the median due to the skewed distribution of ***δ***(*k*). Crucially, ***r***(*k*) was computed using only the *n* × *n* similarity submatrix defined by the *n* individuals comprising the test set of fold *k*. We found that the null hypothesis could be rejected for both the mean eFC benchmark (corrected *t* = 2.47, *p* = 0.008, *df* = 9) and the mean eFC with noise benchmark (corrected *t* = 2.21, *p* = 0.015, *df* = 9).

Finally, we tested whether *f*(***δ***_*pFC*_(*k*) − ***δ***_*meanFC*_(*k*)) significantly exceeded zero for each fold using bootstrapped confidence intervals. This was repeated independently for each fold *k* = 1, …, *K*. The 5% confidence interval exceeded zero for 5 of the 10 folds, indicating that pFC significantly outperformed the mean eFC benchmark for half of the folds. In contrast, mean eFC did not significantly outperform pFC for any folds. The distribution of ***δ***_*pFC*_(*k*) − ***δ***_*meanFC*_(*k*) and the corresponding 5% confidence interval is shown separately for each of the 10 folds in Supporting Information Figure 1C.

### Standardization

Individual functional connectivity can be modelled as variation about the group mean (Rutherford et al., 2023). When correlating FC matrices between individuals, variation in the group mean across connections can potentially overshadow the extent of similarity across individuals. We therefore *z*-scored (standardized across participants) the eFC and pFC matrices before computing the similarity matrix and then repeated testing of the null hypothesis given by Equation 1. The computation of means and standard deviations for *z*-scoring purposes respected the cross-validation folds. We found that standardization of eFC and pFC led to an increase in the difference between interindividual and intraindividual similarity (intra = 0.012, inter = 0, *p* = 0.008, *d* = 0.08; Figure 2B). Interestingly, the null hypothesis could now also be rejected when substituting an individual’s pFC with their SC matrix (intra = 0.0031, inter = 0, *p* = 0.001, *d* = 0.10), but not the null condition (intra = −0.011, inter = 0, *p* = 0.959). Note that SC was also standardized in this case.

**Figure 3.**
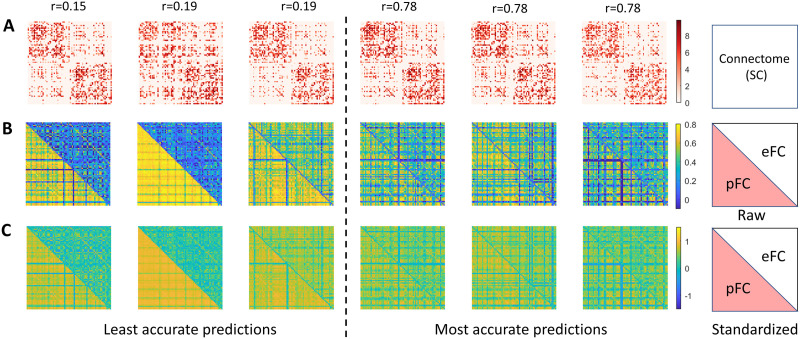
Example individual connectivity matrices. (A) Structural connectome matrices for six individuals. Each column represents an individual. Matrix elements are logarithm transformed interregional streamline counts. (B) Empirical (eFC, upper triangle) and predicted (pFC, lower triangle) functional connectivity matrices for three individuals with the least accurate (three leftmost columns) and most accurate (rightmost) predictions. Correlation coefficients (*r*) between pFC and eFC are indicated for individuals. (C) Same as panel B but for standardized (i.e., *z*-scored) functional connectivity.

Example eFC and pFC matrices are shown for individuals with the least and most accurate predictions in Figure 3. Irregular and somewhat unusual organization is evident for some of the structural connectomes for individuals with the least accurate predictions (e.g., second column, Figure 3A), potentially explaining poor prediction accuracy (see Discussion).

### Riemannian Geometry

Functional connectivity matrices mapped using covariance measures such as correlation are necessarily symmetric and positive semidefinite. Mathematically, such matrices are constrained to a Riemannian manifold. As such, Riemannian distance measures have been considered more appropriate to quantify the difference/similarity between two FC matrices (Dadi et al., 2019; Varoquaux et al., 2010; You & Park, 2021), compared to the more commonly used correlation coefficient and distance measures defined in Euclidean space. We therefore recomputed the similarity matrix using the log-Euclidean Riemannian metric (LERM; Arsigny et al., 2007). LERM embeds points on the Riemannian manifold to Euclidean space via the matrix logarithm (see Methods). We found that the null hypothesis could not be rejected under this alternative geometry (intra = 14.577, inter = 14.637, *p* = 0.073, *df* = 999), although a trend in the hypothesized direction was evident. Note that because LERM is a distance metric, we expect *r*_*inter*_ > *r*_*intra*_.

### Individual Matching

Having found evidence for pFC capturing subtle yet significant individual-specific effects when measuring similarity using correlation-based measures, we next tested whether correlation-based similarity could be used to correctly match an individual’s pFC to their own eFC. This involved solving a binary assignment problem to establish a one-to-one mapping in which similarity was maximized (Figure 1C). Chance-level matching accuracy was established using randomized similarity matrices (see Methods). Matching accuracy depends on sample size. We therefore randomly sampled multiple subsets comprising two to 20 individuals, established a one-to-one pFC-eFC matching for each subset and then measured the proportion of individuals correctly matched as a function of subset size.

**Figure 4.**
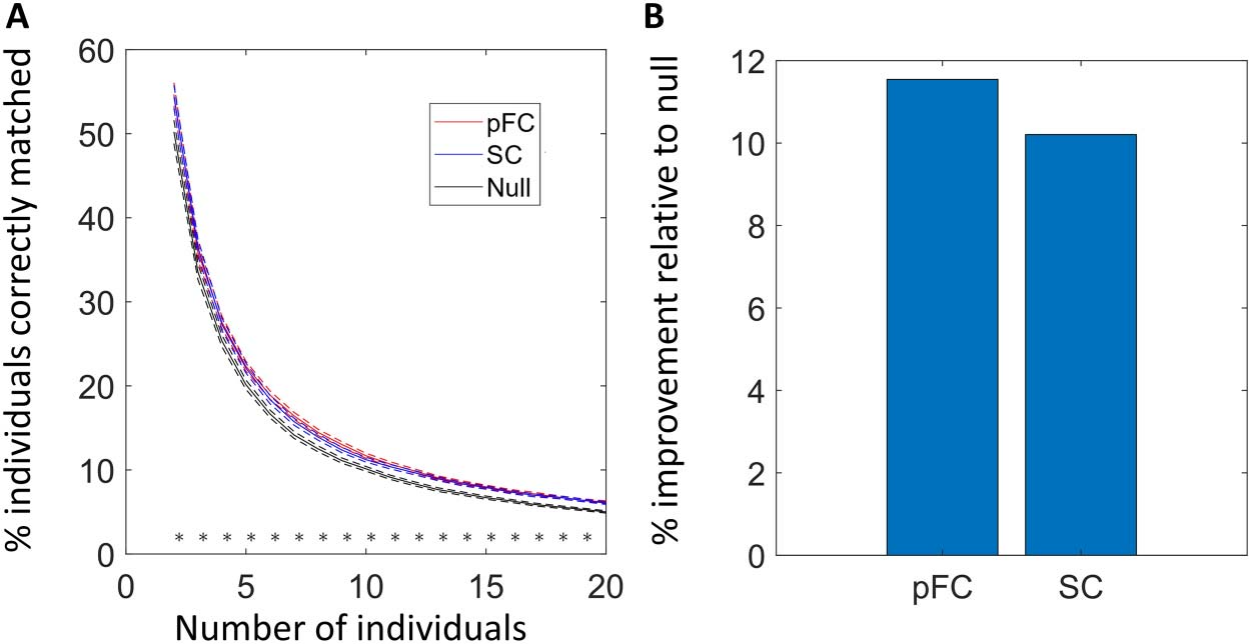
Matching predicted and empirical functional connectivity matrices. (A) Plots show the percentage of individuals correctly identified as a function of the total number of individuals to be matched. Empirical functional connectivity (eFC) is matched to predicted functional connectivity (pFC, red trace), structural connectomes (SC, blue trace), and a null condition (black trace). (B) Bar plots show percentage improvement in matching accuracy relative to null condition. Asterisks denote significant improvement in the percentage of individuals identified compared to the null condition (*p* < 0.05).

We found that pFC and eFC matrices were correctly matched for a significantly greater proportion of individuals than chance levels across multiple subset sizes (Figure 4). For subsets comprising up to 20 individuals, the improvement relative to chance levels was 10.5% ± 2.5%. While statistically significant, this improvement was modest and vanished for sample sizes substantially exceeding the size. Interestingly, we could also match SC and eFC matrices with accuracy significantly above chance levels.

## DISCUSSION

Our work on predicting functional connectivity networks from the structural connectome spurred considerable interest and several follow-up studies (Benkarim et al., 2022; Chen et al., 2023; Hong et al., 2023; Neudorf, Kress, & Borowsky, 2022; Yang et al., 2023). However, concerns have been raised about the extent to which an individual’s predicted functional network captures individual-specific connectivity characteristics beyond group-average effects (Smolders et al., 2023). Here, we revisited our previous individual predictions to investigate these concerns. We proposed measures to assess individual prediction accuracy and applied them to functional network predictions for 1,000 healthy young adults. We found multiple lines of statistically significant evidence supporting the claim that our predicted functional networks successfully capture individual-specific connectivity characteristics that cannot be explained by group-average effects and a null condition. However, effect sizes were generally small, namely, an 8%–11% improvement for both of our measures relative to a group-average benchmark. Thus, caution is needed when drawing conclusions about the individual predictive utility of our and other models. Our findings largely concur with recent work showing that prediction models can capture significant individual-specific effects, although structure-function coupling remains dominated by common network structures found across all individuals (Chen et al., 2023). This is also consistent with earlier work suggesting that functional networks are dominated by common organizational structures with smaller but stable individual effects (Gratton et al., 2018).

Our work is an important advance, given that early investigations suggested *linear* associations between SC and FC unveil limited individual specificity and linear SC-FC coupling is dominated by group effects (Zimmermann et al., 2019). This early work found that an individual’s FC was in most cases not more strongly correlated to its own SC compared to the SC of other individuals. Our work shows that using deep predictive models to capture nonlinear relationships between SC and FC could offer greater sensitivity to subtle individual differences, compared to these early correlational approaches.

While the work of Smolders and colleagues draws critical attention to the importance of evaluating individual prediction accuracy, the results presented here challenge their claim that our and the other prediction models do not provide meaningful individual predictions. The analyses supporting their claims require careful consideration. They considered a control/null prediction that is comparable to the one used here, namely, the group-average eFC matrix with noise added to each matrix element to model individual variation. The group-average benchmark visually appears to yield comparable performance to our predicted functional connectivity, although no statistical testing was undertaken. While group-average effects are indeed prominent, explaining the excellent performance of the group-average benchmark, our statistical testing demonstrates that pFC can significantly (albeit subtly) outperform this benchmark. Additionally, they considered *average* prediction performance, but given that the focus is on *individual* predictions, it would have been more appropriate to consider individual prediction performance; that is, how well does an individual’s pFC match their own eFC, relative to the eFC of other individuals or the group-average eFC?

Smolders and colleagues additionally show that interindividual correlations in eFC and pFC are themselves not significantly correlated with each other. This is an indirect evaluation of prediction performance and the conclusions that can be drawn from this ‘correlation of correlations’ is limited. Interindividual relationships in eFC do not necessarily need to significantly correlate with interindividual relationships in pFC to signify performance exceeding chance levels. The magnitude of interindividual correlations may be relatively consistent among all pairs of individuals, particularly in a homogeneous cohort such as the Human Connectome Project, where the interquartile range of interindividual correlation in eFC is low (i.e., IQR = 0.11). A correlation across these relatively constant correlations will be low. Consider the following geometric counterexample as a demonstration of this effect. Suppose that we aim to predict the three corner coordinates of an equilateral triangle. Each coordinate represents an ‘individual’. Given that our coordinates reside on the Euclidean plane, the Euclidean distance is used to measure the distance between actual and predicted coordinates. Suppose that our prediction is another equilateral triangle centred at the same origin but with a slightly smaller side length. Our prediction outperforms the group average (i.e., origin coordinate) because the distance from the origin to any corner is greater than the distance between corresponding corners of the smaller and larger triangle. However, the distance between each pair of predicted coordinates is the same and equal to the triangle side length. Hence, there is no correlation between predicted and actual interindividual distances.

In our original work, we also demonstrated that an individual’s cognitive performance could be predicted based on their pFC (*r* = 0.29 ± 0.02) or eFC (*r* = 0.33 ± 0.02). We regressed SC from pFC and eFC and used the resulting residuals to train our cognitive prediction model. Smolders and colleagues suggest that regressing SC from pFC may have inadvertently introduced bias and the success of our cognitive prediction model may be driven by SC, not pFC. This is an intriguing suggestion and we do not discount the possibility of SC introducing an inadvertent bias. However, this would need to be studied further and it is important to note that pFC without SC confound regression is significantly associated with cognitive performance, as evaluated with the network-based statistic (see Supporting Information Figure S2). This provides further evidence indicating that pFC captures individual connectivity characteristics relevant to cognition.

### Impacts and Future Development

AI-based prediction of individual functional connectivity is a nascent field. Opportunities to improve prediction accuracy, reliability, scalability, and generalizability of current models are abundant and warrant consideration. First, leveraging recent advances in structural connectome reconstruction is an important avenue for future investigation (Sotiropoulos & Zalesky, 2017). Most work in this area, including our own, has utilized connectomes mapped with basic measures of structural connectivity, such as interregional streamline counts. However, it is well known that streamline counts and connectome mapping in general is hindered by numerous biases and inaccuracies (Maier-Hein et al., 2017). Advanced measures, such as fibre bundle capacity, COMMITT (Daducci et al., 2015) and SIFT (Smith et al., 2015) provide connectivity estimates that better reflect underlying white matter microstructure and may thus enable more durable SC-to-FC mappings to be learnt (Zhang et al., 2018). We observed that poor individual FC predictions were sometimes accompanied by structural connectomes showing unusual characteristics, including a more randomized topological structure and weaker distinction in connection density between intra- and interhemispheric regional pairs (Figure 3). Inaccurate connectome reconstruction could thus potentially explain difficulty in predicting individual connectivity characteristic for these individuals. In the future, it may be possible to exclude structural connectomes that do not pass a quality assurance checks, although this would require development of automated tools to perform such tests. The choice of parcellation atlas may also impact connectome reconstruction accuracy. Previous work suggests that SC-to-FC coupling often weakens with the number of atlas parcels (Seguin, Tian, & Zalesky, 2020). We focused on the Desikan-Killiany atlas here to facilitate explicit comparison with Smolders and colleagues as well as our seminal work.

Second, an important consideration is cohort homogeneity. In our study, model training and evaluation was performed on a relatively homogeneous cohort comprising young, healthy adults (age range: 22–35 years). The extent to which individual effects can be learnt may be limited by the fact that true individual variability is inevitably small across such a narrow age range. Using a more diverse cohort in terms of age range, health status, demographic characteristics, and so forth, would provide greater individual variation for the prediction model to utilize and learn, and thus potentially improve prediction accuracy. Due to the homogeneous cohort studied, our results may underestimate the model performance that could be achieved in a more diverse cohort. Recent efforts towards mapping connectomes for UK Biobank participants (Mansour L. et al., 2023) and other openly available cohorts provides new opportunities to study SC-to-FC mappings in the presence of more representative population diversity than the HCP.

Third, an adequately sized training dataset is required to ensure that sufficient features and dimensions of individual variability can be learnt. Most models in this area have been trained using approximately 1,000 connectomes, giving a feature-to-sample-size ratio of $\frac{68\times 67}{2}\times \frac{1}{1,000}$ = 2.3. In computer vision and natural language processing, comparably deep models are typically trained on datasets comprising tens of thousands to millions of samples. A model may have difficulty predicting a highly unique functional connectivity architecture if it was not provided with comparable training instances. However, as the training sample is increased, it is more likely that genetically related individuals of the same age, sex, and so on, will be encountered, enabling complex and rare patterns to be learnt. The UK Biobank provides high-quality connectomes for approximately 40,000 individuals, and we recommend that future studies utilize this resource.

Finally, the choice of neural network architecture may be consequential. Connectome data is high dimensional and naturally represented as a graph. Work to date has considered either multilayer perceptron architectures or graph convolutional networks and autoencoders (Zhang et al., 2019). Generative models, residual learning and other recent advances in deep learning remain to be applied and could improve model performance. Furthermore, connectomes can now be mapped at ultra-high spatial resolution (Mansour L. et al., 2021), and it will be important to establish scalable architectures that can efficiently handle connectomes comprising 30,000 nodes or more.

The extent to which the above developments and opportunities can improve prediction performance remains to be evaluated. We are optimistic about the prospects of AI-based reconstruction of individual connectomes. Machine learning approaches have already been successfully used to guide white matter tractography (Poulin et al., 2019; Sarwar et al., 2020), and it is perhaps not too much of a leap to envisage functional connectivity estimation based on an individual’s diffusion MRI data.

As the accuracy and reliability of models predicting an individual’s FC from their SC improve, new research and clinical opportunities may emerge. For example, accurate models will enable virtual investigation of functional changes resulting from lesions and connectome pathology, building on recent virtual lesion approaches (Williamson, Greiner, & Kadis, 2023). This may enable clinical researchers to predict in advance the functional consequences of connectome pathology, although this will require training samples featuring pathology. Accurate models may also enable estimation of FC networks for individuals with functional MRI data that is corrupted by head motion or other artifacts. Given the modest effect sizes found here, we contend that significantly improved prediction accuracies would be required to facilitate these opportunities.

At the time of writing this work, the ‘Krakencoder’ tool was released—a deep learning fusion tool that uses a common latent space representation to bidirectionally map between individual SC and FC networks, as well as between different parcellation atlas resolutions and connectome mapping pipelines. The accompanying preprint (Jamison et al., 2024) reports some of the most accurate SC-based predictions of FC achieved to date, significantly outperforming the prediction accuracies of our seminal model (Sarwar et al., 2021) and the Graph Nets architecture (Neudorf, Kress, & Borowsky, 2022), particularly in the fusion mode. The authors assess Krakencoder’s prediction accuracy using metrics akin to those employed here, including demeaned correlation and a binary assignment measure. For example, for a 68-region connectome, our model could correctly assign pFC to eFC for 3% of individuals (chance level: 1%), whereas the Krakencoder fusion mode achieved 9%. Interestingly, the authors found that predicted connectomes correlate more strongly with cognitive scores than empirically mapped connectomes, suggesting that the latent space and connectome fusion can potentially overcome connectome mapping inaccuracies.

### Recommendations

Future studies should statistically evaluate evidence for individual functional connectivity predictions exceeding appropriate benchmark conditions. Here, we proposed two related measures to statistically evaluate individual prediction accuracy and compared performance to a benchmark defined by group-average connectivity. We recommend that future work in this area reports at least one of these measures, or an appropriate alternative measure supported by statistical testing. Differential identifiability (Amico & Goñi, 2018) is an important alternative measure that bears similarity to our first measure, that is, Equation 1. The key difference is that differential identifiability considers whether the *average* of *r*_*intra*_ across all individuals is greater than the *average* of all *N*(*N* − 1)/2 values of *r*_*inter*_. Differential identifiability is thus a two-sample test, whereas our measure is a within-subject design because *r*_*intra*_ is not averaged across individuals. Due to the cross-validation process used to predict pFC, *r*_*intra*_(*i*) is not necessarily independent among individuals *i* = 1, …, *N*, and thus we contend that our within-subject test is more appropriate in these circumstances.

It is also important to report effect size estimates to quantify the extent to which predicted connectivity matrices exceed a null/benchmark condition. Although individual predictions may significantly outperform benchmark conditions, we found that effect sizes were generally small. Reporting the percentage improvement relative to benchmark conditions may also be informative with respect to understanding the magnitude of statistically significant effects.

The choice of distance/similarity measure is an important consideration. Most studies use correlation to quantify similarity between functional connectivity matrices, although Riemann-based distance measures provide a more natural choice for positive-definite matrices (Arsigny et al., 2007). Interestingly, we found that correlation provided stronger and statistically significant evidence compared to a Riemann distance measure. We recommend that future work considers both a correlation-based measure and a measure such as LERM. Graph-based measures could also be used. Of note, eigenmode-based methods were initially found not to outperform the group-average FC prediction (Deslauriers-Gauthier et al., 2020). This can be partly attributed to the minimal interindividual variation in SC across the healthy and homogeneous cohort studied. Interestingly though, using a Riemannian distance (Dadi et al., 2019) between predicted and measured FC to assess prediction accuracy, instead of the usual correlation coefficient, revealed that individual FC predictions significantly outperformed the group-average FC prediction (Deslauriers-Gauthier et al., 2022).

Other key considerations include standardization of functional connectivity before statistical testing and the choice of benchmark/null condition. While these aspects require further investigation, we recommend standardization to better distinguish individual effects from the group average. Standardization should be performed consistent with the cross-validation folds to avoid inadvertent data leakage.

### Conclusions

The evaluations undertaken here suggests that we can predict an individual’s functional connectivity matrix from their structural connectome and our predictions capture subtle individual connectivity effects that are not explained by the group-average connectivity matrix. However, it is important to emphasize that despite their statistical significance, captured individual effects are subtle and overshadowed by the group average. Individual effects may become more evident in future work considering more diverse cohorts, advanced connectome reconstruction techniques or sophisticated deep learning architectures. Larger training samples will also be essential. Indeed, several recent studies following up on our original work have reported significant improvements in prediction accuracy (see Table 1). The ability to match individual differences in structural and functional connectivity lends a degree of validity to established connectome mapping pipelines, despite their well-known limitations (Maier-Hein et al., 2017), although pipeline validation was not a goal of the current study.

Smolders and colleagues contend that it is not possible to ascertain whether our predictions have learnt and meaningful mapping from SC to FC. We have presented multiple lines of evidence to refute this claim. While their work draws much needed attention to the importance of testing the extent to which individual predictions outperform benchmark conditions, their analyses are equivocal, and as they acknowledge, it is not possible to ascertain firm conclusions from their results. It is important that claims and criticisms offered by Smolders and colleagues about other work in this field (i.e., Benkarim et al., 2022; Neudorf, Kress, & Borowsky, 2022) are carefully assessed before conclusions are drawn.

## METHODS

### Connectivity Mapping and Prediction

Resting-state functional connectivity networks, referred to as empirical FC (eFC), and structural connectomes (SC) were mapped for 1,000 healthy adults (age range: 22–35 years) participating in the Human Connectome Project (Van Essen et al., 2013). Connectivity matrices encompassed 68 cortical regions comprising the Desikan-Killiany parcellation atlas (Desikan et al., 2006). The Pearson correlation coefficient was used to infer eFC between all region pairs based on approximately 30 min of minimally processed data (Glasser et al., 2013). To map structural connectomes, constrained spherical deconvolution and whole-brain deterministic tractography was undertaken for the same individuals and parcellations atlas. Whole-brain tractography and connectome mapping was performed with the MRtrix3 package (www.mrtrix.org). Interregional streamline counts were resampled to a normal distribution (Honey et al., 2009). Further methodological details are provided in our earlier publication (Sarwar et al., 2021).

We reused the predicted functional connectivity (pFC) matrices established in our earlier work (Sarwar et al., 2021). In brief, a multilayer perceptron network (eight hidden layers) was trained to learn the mapping from SC to eFC using 10-fold cross-validation across the 1,000 individuals. Each layer comprised 1,024 neurons with a dropout rate of 0.5. Two activation functions were used: leaky rectified linear unit with a leak of 0.2 and the hyperbolic tangent. The objective function was given by, θ* = *l*(*θ*) + *λ*(*γ* − *ϕ*(*θ*)), where *l* is the mean squared error for a network with parameters *θ*, *γ* is the mean interindividual correlation coefficient for eFC in the training set (constant), *ϕ* is the mean interindividual correlation coefficient for pFC, and *λ* is a regularization hyperparameter that was tuned to achieve a trade-off between group-average effects and individual differences. Hyperparameter values and further details are provided in our earlier work (Sarwar et al., 2021).

### Similarity Measures

Similarity/distance measures were used to quantify the distance between pairs of functional connectivity matrices. To compute the Pearson correlation coefficient, the upper triangular elements of both matrices were vectorized and the usual Pearson correlation coefficient was computed. This was repeated for all pairs of individuals to populate a similarity matrix. The log-Euclidean Riemannian metric (LERM) between FC matrices *A* and *B* was computed as,

$${\text{LERM}}^{2}={\left\|Log\left(A\right)-Log\left(B\right)\right\|}_{F}^{2}.$$

In this formulation of LERM, ${\left\|\cdot \right\|}_{F}^{2}$ denotes the Frobenius norm and *Log*(·) is the matrix logarithm. We also considered the affine-invariant Riemann metric (AIRM), which characterizes intrinsic geometry on the space of semipositive definite matrices (Arsigny et al., 2007). However, AIRM was computationally intractable for the dimensionalities considered here due to a costly matrix inversion, and results are not reported.

### Binary Assignment

Individual eFC-pFC assignments were performed with the Hungarian algorithm (Kuhn, 1955). The Hungarian algorithm was used to derive a one-to-one mapping between individual eFC and pFC matrices. If we had instead sequentially assigned each eFC matrix to the pFC matrix with which it is most strongly correlated, one-to-many assignments would be permitted in which multiple individuals are assigned to the same matrix. (Note that a one-to-many assignment may nonetheless yield a reasonable measure of matching accuracy.) We first randomly sampled (without replacement) a subset of *n* < *N* individuals and computed a corresponding *n* × *n* correlation-based similarity matrix. The similarity matrix was negated to ensure that the Hungarian algorithm penalized eFC-pFC matchings between individuals with dissimilar connectivity matrices. The Hungarian algorithm transformed the similarity matrix into a binary assignment matrix comprising exactly one nonzero entry for each row and column. The proportion of individuals matched correctly was given by normalizing the trace of the assignment matrix by *n*. For *n* = 2, 3, …, 20, this process was independently repeated for *M* = 2,500 random subsets to compute an average matching accuracy for each value of *n*. Samples within a given subsets were always drawn from the same cross-validation fold. Chance-level matching accuracy was established using randomized similarity matrices. Specifically, columns (or rows) of the similarity matrix were randomly permuted, after which the Hungarian algorithm was applied to compute a matching consistent with chance levels, providing a null condition for assignment matching accuracy. To establish chance expectations, we also considered similarity matrices derived from a Gaussian matrix in which each element was independently sampled from a Gaussian distribution. These two randomization methods yielded comparable findings and here we only report findings for the former methodology.

### Statistical Testing

The null hypothesis specified by Equation 1 was assessed using multiple approaches. *Naïve method*: We first conducted a one-tailed, one-sample *t* test (*df* = 999). This test does not account for potential statistical dependencies induced by the cross-validation process. *Permutation testing*: Permutation testing was used to empirically generate a null distribution. Permutations were constrained to occur within folds, but never across folds (Supporting Information Figure S1A), thereby preserving interfold dependencies. For each permutation, a one-tailed, one-sample *t* test was used to quantify the difference between *r*_*intra*_ and *r*_*inter*_. This ensured that a pivotal statistic was used to generate the null distribution. *Corrected resampled t test statistic*: We considered the corrected resampled *t* test statistic (Nadeau & Bengio, 2003), given by,

$$t_{\text{corrected}}=\frac{1/K\sum_{k=1,\ldots ,K}f\left(\delta_{pFC}\left(k\right)-\delta_{\text{meanFC}}\left(k\right)\right)}{\sqrt{\frac{1}{K}+\frac{n}{n_{1}}{\hat{\sigma }}^{2}}}∼t_{K-1}$$

In this formulation, *n* is the test set sample size (100), *n*_1_ is the training sample size (900), *K* is the number of cross-validation folds (10), *f*(·) is a central tendency measure (mean or median), and ***δ***_*pFC*_(*k*) − ***δ***_*meanFC*_(*k*) ∈ *R*^*n*×1^ is a measure of the difference in prediction performance between pFC and the mean eFC benchmark for each of the *n* individuals in fold *k*. We measured model performance as ***δ***(*k*) = ***r***_*intra*_(*k*) − ***r***_*inter*_(*k*), where ***r***(*k*) was computed using only the *n* × *n* similarity submatrix defined by the *n* individuals comprising the test set of fold *k*. This measure of model performance quantifies the compromise between intra- and interindividual similarity. The variance term ${\hat{\sigma }}^{2}$ in the above formulation was estimated across the *k* = 1, …, *K* samples of *f*(***δ***_*pFC*_(*k*) − ***δ***_*meanFC*_(*k*)). The variance terms could alternatively be estimated with bootstrapping. Note that that naïve method and permutation testing consider a *within-model comparison* (i.e., *r*_*intra*_ vs. *r*_*inter*_), whereas the corrected *t* test considers a *between-model comparison* (i.e., pFC vs. benchmark). In all cases, one-tailed tests were conducted, given the strong rationale for a directional alternative hypothesis (i.e., *r*_*intra*_ > *r*_*inter*_). A *p* value less than 0.05 was deemed statistically significant for all tests.

For the individual matching measure, a two-sample *t* test (equal variance, *df* = *m* × 2 − 2) was used to assess for differences in the proportion of individuals correctly matched between pFC and the null condition. The false discovery rate (FDR) was controlled at 5% across the set of 19 sample sizes evaluated (i.e., *n* = 2, 3, … 20). Note that the samples were always drawn from the same cross-validation fold to avoid between-fold dependency effects.

### Code and Data Availability

- MATLAB code implementing the tests described here: https://github.com/AndrewZalesky/SC-FC-prediction
- LERM and AIRM were computed using the SPDtoolbox library: https://github.com/kisungyou/papers/tree/master/01-SPDtoolbox
- Hungarian algorithm implementation: https://au.mathworks.com/matlabcentral/fileexchange/20328-munkres-assignment-algorithm
- Neural network architecture and example SC and eFC matrices: https://github.com/sarwart/mapping_SC_FC
- HCP neuroimaging data are available for download from the ConnectomeDB platform: www.humanconnectome.org

## ACKNOWLEDGMENTS

Data were provided by the Human Connectome Project, WU-Minn Consortium (Principal Investigators: David Van Essen and Kamil Ugurbil; 1U54MH091657) funded by the 16 NIH Institutes and Centers that support the NIH Blueprint for Neuroscience Research; and by the McDonnell Center for Systems Neuroscience at Washington University. BTTY is supported by the NUS Yong Loo Lin School of Medicine (NUHSRO/2020/124/TMR/LOA), the Singapore National Medical Research Council (NMRC) LCG (OFLCG19May-0035), NMRC CTG-IIT (CTGIIT23jan-0001), NMRC OF-IRG (OFIRG24jan-0030), NMRC STaR (STaR20nov-0003), Singapore Ministry of Health (MOH) Centre Grant (CG21APR1009), the Temasek Foundation (TF2223-IMH-01), and the United States National Institutes of Health (R01MH133334). Any opinions, findings and conclusions or recommendations expressed in this material are those of the authors and do not reflect the views of the funders.

## SUPPORTING INFORMATION

Supporting information for this article is available at https://doi.org/10.1162/netn_a_00400.

## AUTHOR CONTRIBUTIONS

Andrew Zalesky: Conceptualization; Data curation; Formal analysis; Funding acquisition; Investigation; Methodology; Project administration; Resources; Software; Supervision; Validation; Visualization; Writing – original draft; Writing – review & editing. Tabinda Sarwar: Conceptualization; Data curation; Formal analysis; Funding acquisition; Investigation; Methodology; Project administration; Resources; Software; Supervision; Validation; Visualization; Writing – original draft; Writing – review & editing. Ye Tian: Conceptualization; Data curation; Formal analysis; Funding acquisition; Investigation; Methodology; Project administration; Resources; Software; Supervision; Validation; Visualization; Writing – original draft; Writing – review & editing. Yuanzhe Liu: Conceptualization; Data curation; Formal analysis; Funding acquisition; Investigation; Methodology; Project administration; Resources; Software; Supervision; Validation; Visualization; Writing – original draft; Writing – review & editing. B. T. Thomas Yeo: Conceptualization; Data curation; Formal analysis; Funding acquisition; Investigation; Methodology; Project administration; Resources; Software; Supervision; Validation; Visualization; Writing – original draft; Writing – review & editing. Kotagiri Ramamohanarao: Conceptualization; Data curation; Formal analysis; Funding acquisition; Investigation; Methodology; Project administration; Resources; Software; Supervision; Validation; Visualization; Writing – original draft; Writing – review & editing.

## FUNDING INFORMATION

AZ is supported by the ARC Future Fellowship and Rebecca L. Cooper Foundation. YT is support by the NHMRC Emerging Leadership Fellowship and Mary Lugton Fellowship. YL is supported by a Melbourne Research Scholarship.

## Supplementary Material



## TECHNICAL TERMS

Structural connectome (SC): Network representation of structural connections derived from diffusion MRI and white matter tractography.
Empirical functional connectivity (eFC): Temporal correlations between spatially distant brain regions, inferred from resting-state functional MRI.
Predicted functional connectivity (pFC): Functional connectivity predicted from an individual’s structural connectome matrix, in the absence of any functional data.
Interindividual similarity: The extent to which functional brain networks are similar between distinct individuals, assessed using the correlation coefficient.
Riemannian distance: A distance measure that is well suited to measuring distances on curved spaces, such as the space spanned by functional connectivity matrices.
Differential identifiability: A measure to determine the extent to which an individual’s brain network can be distinguished from those of other individuals.
Hungarian algorithm: An efficient and exact method to find a one-to-one matching between two sets of objects, subject to minimizing a cost function.
