# Supplementary material for: Predicting an individual’s functional connectivity from their structural connectome: Evaluation of evidence, recommendations, and future prospects
Source: Netw Neurosci. 2024 Dec 10;8(4):1291–309. doi: 10.1162/netn_a_00400 (PMC11674402; doi:10.1162/netn_a_00400)
Supplement: Supplementary file 1 [file netn-8-4-1291-s001.pdf]

Predicting an individual's functional connectivity from their structural connectome: Evaluation of evidence, recommendations and future prospects

Andrew Zalesky[1,2], Tabinda Sarwar[3], Ye Tian[1], Yuanzhe Liu[1], B.T. Thomas Yeo[4], Kotagiri Ramamohanarao[5]

1. Systems Lab, Department of Psychiatry, The University of Melbourne, Victoria 3010, Australia
2. Department of Biomedical Engineering, The University of Melbourne, Victoria 3010, Australia
3. School of Computing Technologies, RMIT University, Victoria, 3000, Australia
4. Department of Electrical and Computer Engineering, Center for Sleep & Cognition & N.1 Institute for Health, National University of Singapore, 15 119077, Singapore
5. Retired Professor, The University of Melbourne, Victoria 3010, Australia

To whom correspondence should be addressed:

Dr Andrew Zalesky
Systems Lab, Departments of Psychiatry & Biomedical Engineering
Level 3, Alan Gilbert Building
The University of Melbourne, Victoria 3010, Australia
Email: azalesky@unimelb.edu.au

Supplementary Figures

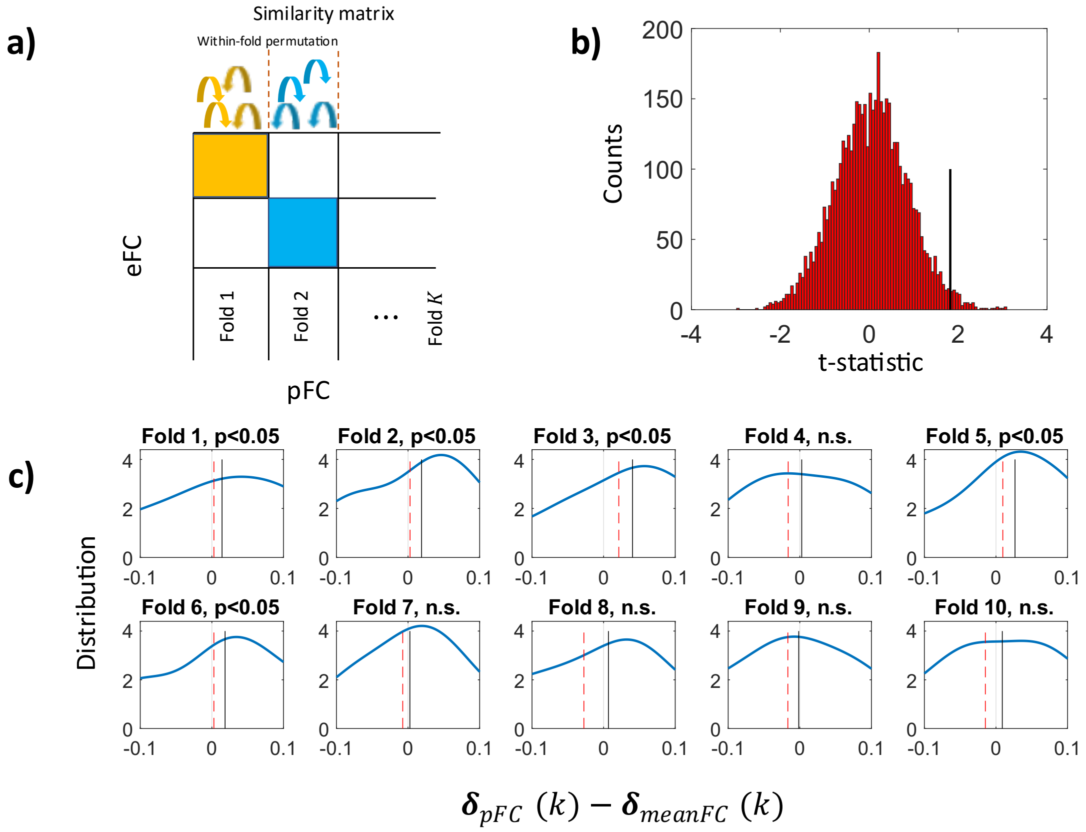

Supplementary Figure 1. **(a)** Visualization of similarity matrix permutation. Columns of the similarity matrix were permuted within individuals comprising the same cross-validation fold. Columns were never permitted between two distinct folds. **(b)** Histogram shows the empirical null distribution generated with permutation. Each permutation generated a t-statistic quantifying the extent to which the set of differences $r_{intra}(i) - r_{inter}(i), i = 1, \ldots, N$, deviated from zero in the permuted data. Solid vertical black line indicates the corresponding t-statistic for the observed data. **(c)** Histograms show the distribution of differences in prediction accuracies between pFC and the mean eFC benchmark. A separate distribution is shown for each cross-validation fold, $k = 1, \ldots, K$. Solid vertical black line indicates distribution median. Dashed vertical red line indicates 5% confidence interval. If the 5% confidence interval falls above zero, pFC was deemed to significantly outperform the benchmark prediction. n.s. not significant. The benchmark prediction never significantly outperformed pFC.

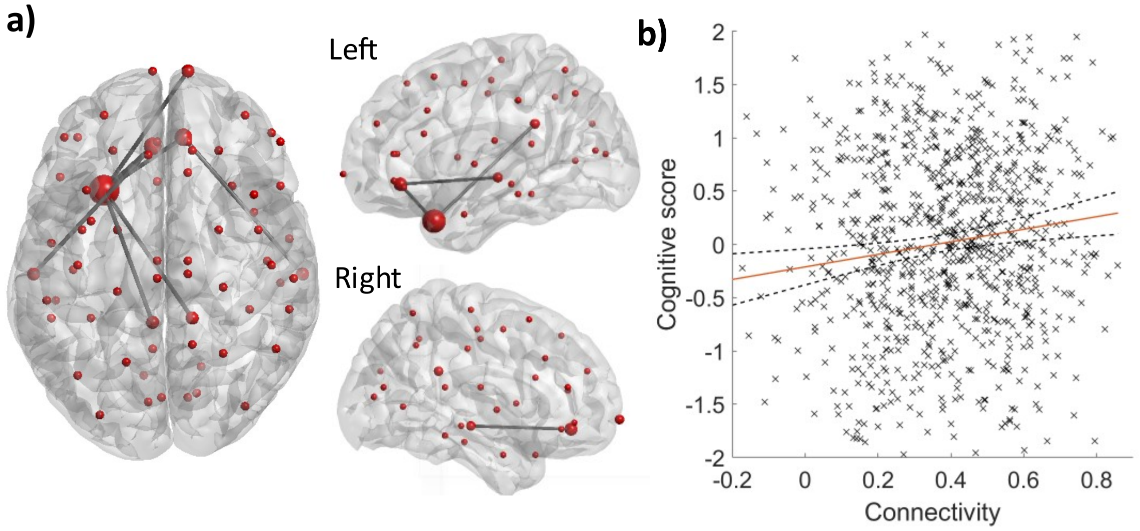

Supplementary Figure 2. (a) Network visualization shows subnetwork of predicted functional connections (pFC) significantly associated with cognitive performance (p=0.033). The subnetwork was identified using the network-based statistic (default primary threshold: t-stat=3.1, 5000 permutations). Cognitive data was available for 940 individuals, as described elsewhere (Tian et al. 2020). Unlike our earlier work (Sarwar et al. 2021), SC was not regressed from pFC. **(b)** Scatter plot shows correlation between cognitive scores and average functional connectivity across the subnetwork (r=0.12). Dashed lines indicate 95% confidence intervals.
